# Elemental, fatty acid, and protein composition of appendicoliths

**DOI:** 10.1038/s41598-022-21397-9

**Published:** 2022-11-17

**Authors:** James M. Prieto, Andrew W. Wang, Jonathan Halbach, David M. Cauvi, James M. D. Day, Milan Gembicky, Majid Ghassemian, Oswald Quehenberger, Karen Kling, Romeo Ignacio, Antonio DeMaio, Stephen W. Bickler

**Affiliations:** 1 Division of Pediatric Surgery, Rady Children’s Hospital, San Diego, CA USA; 2 Naval Medical Center San Diego, San Diego, CA USA; 3 Department of Surgery, School of Medicine, University of California San Diego, 9500 Gilman Drive #0739, La Jolla, CA 92093-0739 USA; 4 Scripps Institution of Oceanography, University of California San Diego, La Jolla, CA USA; 5 Crystallography Facility, Department of Chemistry & Biochemistry, University of California San Diego, La Jolla, CA USA; 6 Biomolecular and Proteomics Mass Spectrometry Facility, University of California San Diego, La Jolla, CA USA; 7 Lipidomics Core, University of California San Diego, La Jolla, CA USA; 8 Center for Investigations of Health and Education Disparities, University of California San Diego, La Jolla, CA USA

**Keywords:** Gastroenterology, Medical research, Molecular medicine, Pathogenesis, Risk factors

## Abstract

Appendicoliths are commonly found obstructing the lumen of the appendix at the time of appendectomy. To identify factors that might contribute to their formation we investigated the composition of appendicoliths using laser ablation inductively coupled plasma mass spectroscopy, gas chromatography, polarized light microscopy, X-ray crystallography and protein mass spectroscopy. Forty-eight elements, 32 fatty acids and 109 human proteins were identified within the appendicoliths. The most common elements found in appendicoliths are calcium and phosphorus, 11.0 ± 6.0 and 8.2 ± 4.2% weight, respectively. Palmitic acid (29.7%) and stearate (21.3%) are the most common fatty acids. Some stearate is found in crystalline form—identifiable by polarized light microscopy and confirmable by X-ray crystallography. Appendicoliths have an increased ratio of omega-6 to omega-3 fatty acids (ratio 22:1). Analysis of 16 proteins common to the appendicoliths analyzed showed antioxidant activity and neutrophil functions (e.g. activation and degranulation) to be the most highly enriched pathways. Considered together, these preliminary findings suggest oxidative stress may have a role in appendicolith formation. Further research is needed to determine how dietary factors such as omega-6 fatty acids and food additives, redox-active metals and the intestinal microbiome interact with genetic factors to predispose to appendicolith formation.

## Introduction

Appendicitis is the most common abdominal surgical emergency in children, with a lifetime risk of about nine percent^1^. In 2019, there were approximately 133,000 cases of appendicitis in the United States in the under 20 years age group^2^. While the principles of surgical management of appendicitis are well established, there is no consensus on the cause(s) of appendicitis more than 130 years after Reginald Fitz’s first description of the condition in 1886^3–5^.

**Figure 1:**
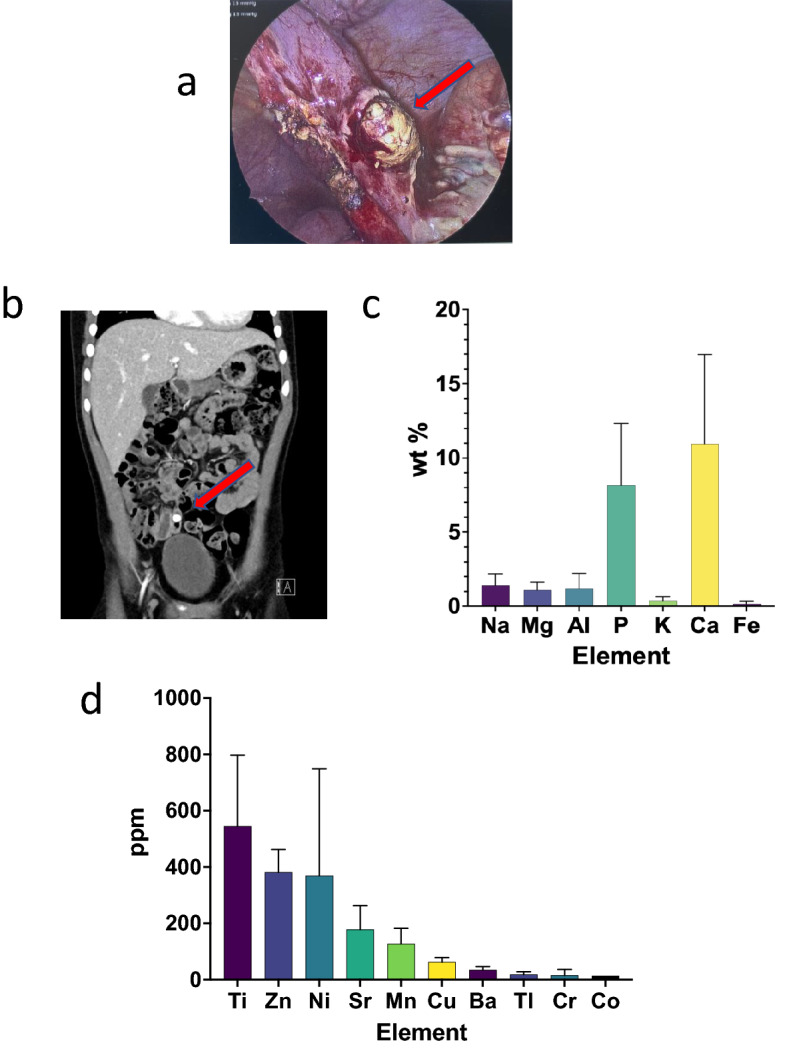
Appearance and elemental composition of appendicoliths (**a**) Intra-operative photo of appendicolith eroding through wall of the appendix (Source: author photo). (**b**) Computerized tomography scan of the abdomen showing an appendicolith in the right lower quadrant of the abdomen (Source: author photo). (**c**) Most common elements found in appendicoliths. (**d**) Ten most common trace elements found in appendicoliths.

Historically, obstruction of the lumen of the appendix has been thought to have an important role in appendicitis^6^, resulting in accumulation of intraluminal mucus, dilation, venous hypertension and ultimately tissue ischemia that can culminate with perforation. In approximately half of all patients, the inciting obstruction is an appendiceal concretion (Fig. 1a,b), most commonly referred to as a fecalith, or appendicolith^7^. As appendicitis can occur in the absence of an appendicolith, obstruction of the lumen is not likely to completely explain the pathogenesis of the disease.

Despite the common nature of appendicoliths, there is a paucity of information on their composition. In 1907, O.T. Williams observed appendix concretions contained large quantities of fat and calcium^8^. Williams et al. compared the fatty acid composition of feces and appendicoliths in 1960, and reported that appendicoliths contain a predominance of palmitic and stearic acids^9^. The most recent study of appendicolith composition we found in a literature review was by López Vásquez et al. in 1980, who used microscopy and infrared spectrophotometry to show that appendicoliths are abundant in-organic material as well as organic acids and hydrocarbons^10^. The objective of this study was to apply modern physical sciences analytical techniques to appendicoliths, to gain insight into possible mechanisms that lead to their formation.

## Methods

### Specimen collection

Five appendicoliths were collected from children undergoing appendectomy for acute appendicitis at Rady Children’s Hospital in San Diego, California. The appendicoliths were separated from the surgical specimen, de-identified and stored at − 80 °C until analysis. The research was approved by the UCSD Human Research Protections Program and performed in accordance with relevant guidelines/regulations. Informed consent was obtained from a parent and/or legal guardian.

### Element composition

Elemental ratios within appendicoliths were determined by laser ablation inductively coupled plasma mass spectrometry (LA-ICP-MS) at the Scripps Isotope Geochemistry Laboratory, using a *New Wave Research* UP213 (213 nm) laser-ablation system coupled to a *ThermoScientific* iCAPq C ICP-MS. Analyses were done using a 100 μm beam diameter, a laser repetition rate of 5 Hz, and a photon fluence of 3.5 J/cm^2^ in a 3 cm^3^ ablation cell. The cell was flushed with He-gas to enhance production and transport of fine aerosols and the ablated sample was mixed with an Ar carrier-gas flow of ~ 1 L/min before reaching the torch. Each analysis consisted of ~ 60 s of data collection. Backgrounds on the sample gas were collected for ~ 20 s followed by ~ 40 s of laser ablation. Washout time between measurements was > 120 s.

The following masses were monitored during analysis: 7Li, 10B, 23Na, 24Mg, 27Al, 29Si, 31P, 35Cl, 39K, 44Ca, 48Ti, 55Mn, 57Fe, 63Cu, 66Zn, 77Se, 88Sr, 133Cs, 137Ba, 139La, 140Ce, 141Pr, 146Nd, 147Sm, 153Eu, 157Gd, 159Tb, 163Dy, 165Ho, 166Er, 169Tm, 172Yb, 204Pb, 206Pb, 207Pb, 208Pb and 238U. Data were collected in time-resolved mode. Plots of counts per second versus time were examined for each analysis, and integration intervals for the gas background and the sample analysis were selected. The NIST 610 glass and BHVO-2g standards were measured for internal calibration and to correct for laser-induced element fractionation.

### Crystallography

A small amount of each appendicolith sample was individually cut from the parent sample and visually inspected under a microscope using transmitted polarized light (magnification 10–50×). From heterogeneous samples, small domains of crystals were selected (approximate size 0.25 × 0.20 × 0.20 mm^3^) and mounted on a polyester loop appropriate for protein crystallography. XRD data were collected on a four-circle diffractometer using a Vantec 500 detector (Bruker, Madison, Wisconsin) equipped with Cu Kα radiation (l = 1.5478) at the UCSD X-ray Crystallography Facility. Diffraction images were merged/integrated into EVA software (Bruker, Madison, Wisconsin) and the peak positions were compared to the calculated patterns for known crystal structures to confirm sample identity.

### Fatty-acid content

Fatty acids were analyzed by the LIPID MAPS UCSD Lipidomics Core as previously described^11,12^. For total fatty acid analysis, five mg of each appendicolith sample were extracted by a modified Bligh and Dyer method. To compensate for potential losses during sample preparation, a cocktail of deuterated internal fatty acid standards was added before the extraction procedure. The lipid extract was hydrolyzed with 0.5 N potassium hydroxide for 1 h at 37 °C. The fatty acid hydrolysate was then brought to neutral pH by addition of hydrochloric acid and the free fatty acids were extracted with isooctane. The combined isooctane layers were evaporated to dryness under argon. The extracted free fatty acids were derivatized with pentafluorobenzyl bromide and the fatty acid esters were analyzed by GC/MS on an Agilent 6890 N gas chromatograph equipped with an Agilent 5973 mass selective detector (Agilent, Santa Clara, CA). Fatty acid quantitation was achieved by the stable isotope dilution method using concentration curves generated from fatty acid quantitative standards using identical conditions. Quantification was done using Microsoft Excel Version 15.32.

### Protein mass spectroscopy

Intact appendicolith samples were cut into two-by-two mm cubes and washed three times in 1 ml phosphate saline buffer to remove any trace of blood from the samples. Samples were then ground in liquid nitrogen to powder. Six molar Guanidine solution (10 × volume of the pellet) was added to the ground pellet and vortexed to mix. The sample was then boiled for 5 min followed by 5 min of vigorous vortexing for three cycles. The samples were then centrifuged on the desktop microfuge at 15,000 rpm for 5 min. The supernatant was transferred to a fresh tube and ten volumes of methanol added. The samples were mixed and allowed to sit at room temperature for 5 min then centrifuged at 15,000 rpm for 15 min. The liquid was removed, and the pellet resuspended in 8 M Urea made in 100 mM Tris pH 8.0 (100 ug protein with 50 ul of eight molar urea solution). TCEP (tris(2-carboxyethyl)phosphine) was added to the solution to a final concentration of 2 mM. Chloro-acetamide solution was then added to a 40 mM final concentration. The solution was then diluted using 50 mM tris pH 8.0 so the final concentration of urea was 2 M. Trypsin (1:50 ug ratio of trypsin: protein) was added to the sample, with overnight digestion on a shaking plate. The following day the solution was acidified using Trifluoroacetic acid (TFA) to a final of 0.5% TFA. The samples were then centrifuged on the desktop microfuge at 15,000 rpm for 15 min to remove the insoluble. The peptides in the solution were purified using C18 tips (Pierce™ C18 Tips, 100 µL bed 87,784) as suggested by the manufacturer protocol.

Liquid chromatography coupled with tandem mass spectrometry was carried out as follows: Trypsin-digested peptides were analyzed by ultra-high pressure liquid chromatography (UPLC) coupled with tandem mass spectroscopy (LC–MS/MS) using nano-spray ionization. The nanospray ionization experiments were performed using an Orbitrap Fusion Lumos hybrid mass spectrometer (ABSCIEX) interfaced with nanoscale reversed-phase UPLC (Waters corporation nano ACQUITY) using a 25 cm, 75-micron ID glass capillary packed with 1.7-µm C18 (130) BEHTM beads (Waters corporation). Peptides were eluted from the C18 column into the mass spectrometer using a linear gradient (5–80%) of ACN (Acetonitrile) at a flow rate of 375 μl/min for 1 h. The buffers used to create the ACN gradient were Buffer A (98% H2O, 2% ACN, 0.1% formic acid) and Buffer B (100% ACN, 0.1% formic acid). Mass spectrometer parameters are as follows: an MS1 survey scan using the orbitrap detector (mass range (m/z): 400–1500 (using quadrupole isolation), 120,000 resolution setting, spray voltage of 2200 V, Ion transfer tube temperature of 275 C, AGC target of 400,000 and maximum injection time of 50 ms) were followed by data-dependent scans (top speed for most intense ions, with charge state set to only include + 2 − 5 ions, and 5 s exclusion time, while selecting ions with minimal intensities of 50,000 at in which the collision event was carried out in the high energy collision cell (HCD Collision Energy of 30%), and the fragment masses were analyzed in the ion trap mass analyzer (with ion trap scan rate of turbo, first mass m/z was 100, AGC Target 5000 and maximum injection time of 35 ms).

### Statistical analyses

Unless otherwise specified results were analyzed using Microsoft Excel version 16.56, with results expressed as Mean ± SD. For analysis of mass spectroscopy data, proteins were converted into their corresponding gene symbols using MyGene^13,14^. Enrichment analysis of the genes encoding the proteins common to all five appendicolith specimens was done using TopGene^15^.

## Results

Forty-eight elements, 32 fatty acids and 109 human proteins were identified within the appendicoliths. Calcium and phosphorus were the most common elements, comprising 11.0 ± 6.0% and 8.2 ± 4.2% by weight, respectively. Other common elements include sodium, aluminum, magnesium, potassium and iron (Fig. 1c). The ten most common trace elements are shown in Fig. 1d, with titanium (Ti), zinc (Zn), nickel (Ni) and strontium (Sr) being the four most common trace elements identified.

**Figure 2:**
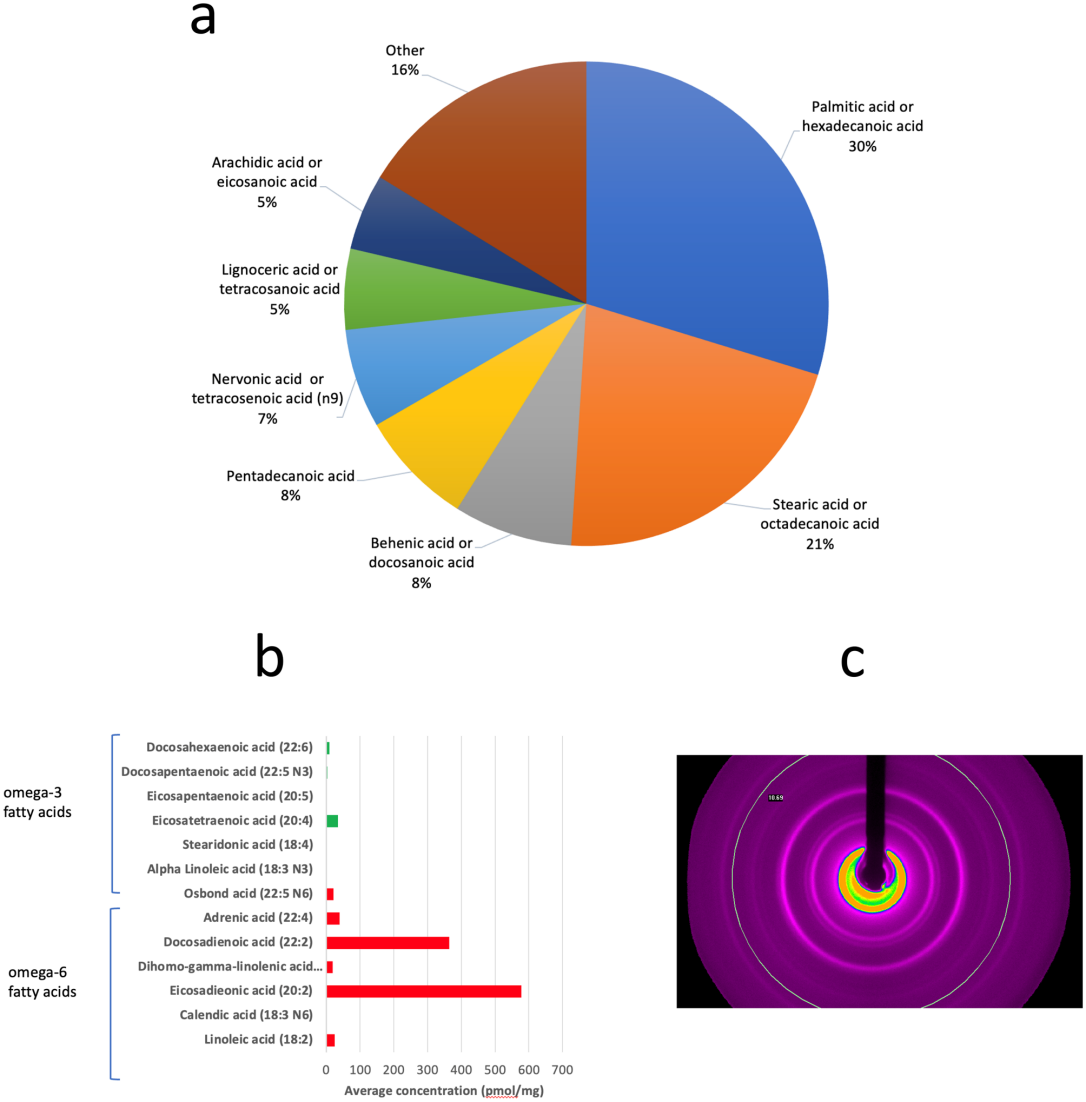
Fatty acid composition of appendicoliths. (**a**)Distribution of fatty acids. (**b**)Omega-3 and omega-6 fatty acids found in appendicoliths. (**c**)Refraction pattern of a stearate crystal identified within an appendicolith using polarized light microscopy.

The most abundant fatty acids in appendicoliths are palmitic acid (29.7%) and stearic acid (21.3%) (Fig. 2a). Appendicoliths also have an increased ratio of omega-6 to omega-3 fatty acids (Fig. 2b). The omega-6 fatty acids with the greatest average concentration were eicosadienoic acid (20:2) and docosadienoic acid (22:2). The ratio of total omega-6 to omega-3 fatty acids was 22:1. Examination of the appendicoliths with polarized light microscopy revealed polycrystalline domains with an appearance like solidified/crystalized oils and having a diffraction pattern consistent with stearate (Fig. 2c).

**Figure 3:**
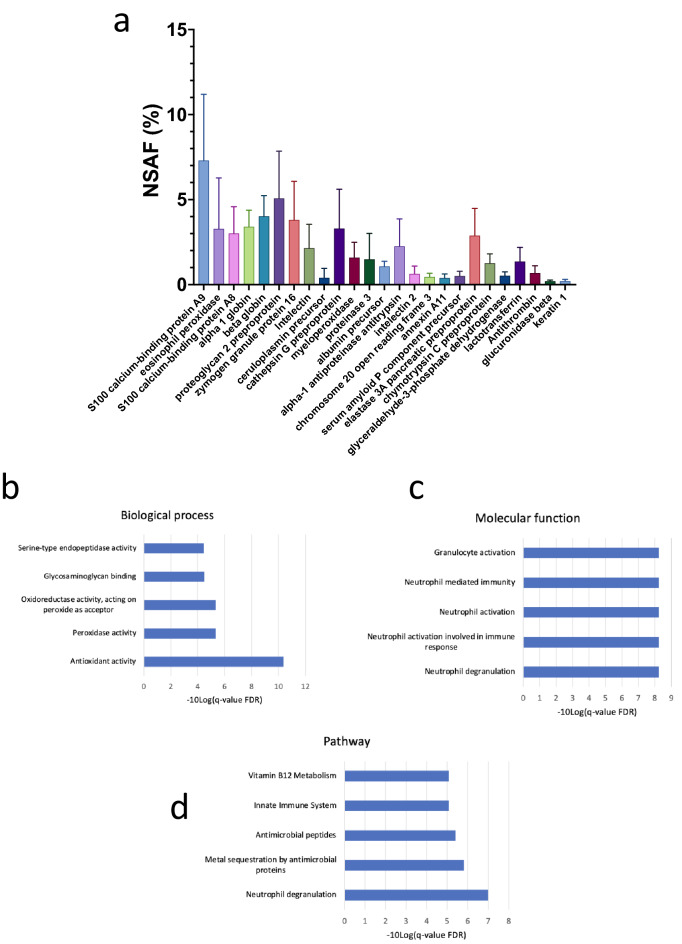
Protein composition of appendicoliths. (**a**) Proteins common to the five appendicoliths analyzed. (**b**) Biological processes identified by ToppGene. (**c**) Molecular functions identified by ToppGene. (**d**) Pathways identified by ToppGene.

Appendicoliths were found to contain both human and bacterial proteins. Of the 249 distinct proteins identified, 140 were of human origin and 109 were produced by gut bacteria. Sixteen human proteins were common to all the appendicolith specimens (Fig. 3a). The most abundant common protein was S-100 calcium-binding protein A9. Analysis of the 16 genes encoding the proteins found in all five appendicoliths showed enrichment of the antioxidant activity biological process (Fig. 3b, Supplementary Table 1a), and the neutrophil activation and degranulation pathways (Fig. 3c,d, Supplementary Table 1b,c).

## Discussion

In this study, we used state-of-the-art analytical tools to investigate the composition of appendicoliths, which are commonly found obstructing the lumen of the appendix at the time of appendectomy. Our analysis of five appendicoliths collected from children undergoing appendectomy for acute appendicitis revealed several interesting findings.

First, we found that palmitic acid and stearate are the most common fatty acids within appendicoliths. Palmitic acid is the most common saturated fatty acid found in the human body and can be provided in the diet, or synthesized endogenously from other fatty acids, carbohydrates and amino acids^16^. Full fat cheeses, red meat, butter, corn oil and palm oil (frequently added to processed foods) contain large amounts of palmitic acid^17^. Interestingly, palmitic acid has been identified as a major component of gallstones, and diets high in palmitic acid have been associated with increased gallstone formation in animal models^18,19^. Stearate, in contrast, is a fatty acid food additive, typically present as a magnesium, sodium, zinc, or calcium salt compound, that is nearly insoluble in water^20^. It has multiple industrial applications including as an additive in soaps, rubber, plastics, and concrete. It is a common ingredient in pharmaceutical tablets, candy, chewing gum, and baked goods, and can be added for anticaking, emulsifying, and binding properties. Of note, was that some of the stearate within appendicoliths exists in crystalline form—identified by polarized light microscopy and confirmed by X-ray crystallography. Understanding more about the chemical composition of these crystals, the environment which allows them to form, and their physiological consequence should be an area of future research. Whether dietary palmitic acid and stearate are a primary cause of appendicoliths or if they accumulate within the lumen of the appendix because of diminished gastrointestinal motility is also unknown.

Second, we observed that there is an increased ratio of omega-6 to omega-3 fatty acids within appendicoliths. This may be of clinical importance as dietary fatty acids have a role in mediating gastrointestinal inflammation^21–23^ and have been linked to inflammatory bowel disease and colorectal cancer^24,25^. Importantly, an elevated ratio of omega-6 to omega-3 fatty acids in Western diets has been hypothesized to be a cause of the relatively high rates of inflammatory conditions, cardiovascular disease, obesity and cancer in high-income countries^26^. In our analysis, two omega-6 fatty acids, docosatetraenoic acid and eicosadienoic acid were the most elevated. Docosatetraenoic acid, also known as adrenic acid, is a naturally occurring polyunsaturated fatty acid (PUFA) formed through a 2-carbon chain elongation of arachidonic acid^27^. Eicosadienoic acid (Δ11, 14–20:2; EDA) is a rare, naturally occurring n-6 PUFA found mainly in animal tissues^28^. EDA is elongated from linoleic acid (LA and can also be metabolized to dihomo-γ-linolenic acid (DGLA), arachidonic acid (AA) and sciadonic acid (Δ5, 11, 14–20:3; SCA). Of note, when macrophages are exposed to lipopolysaccharide (LPS), EDA decreases the production of nitric oxide (NO) and increases the production of prostaglandin E(2) (PGE(2)) and tumor necrosis factor-α^29^. Whether omega-6 fatty acids, specifically docosatetraenoic acid and eicosadienoic acid, have a role in appendicolith formation and in acute appendicitis will require further study.

Third, we discovered that appendicoliths contain a distinct pattern of minerals and metals. Calcium and phosphorus are the most common elements found in appendicoliths, the former likely explaining their radiopaque nature (Fig. 1b). As the calcium occurs in approximately the same ratio as the phosphorus, we suspect it likely occurs as calcium phosphate—a family of minerals containing calcium ions (Ca^2+^) together with inorganic phosphate anions. Unexpectedly, we also found multiple other metals present in appendicoliths. Some of these metals are redox-active, especially manganese, iron and zinc. As such, they could potentially undergo redox cycling reactions resulting in the production of reactive oxygen/nitrogen species (RONS). For example, in Fenton’s reaction, iron reacts with hydrogen peroxide to produce hydroxyl free radicals, one of the most reactive RONS molecules^30^. Excess intracellular RONS can disrupt the intracellular redox state and energy production resulting in oxidative stress, which manifests itself in the modification of cellular biomolecules, such as DNA, lipids and proteins, the dysfunction of mitochondrial respiration, protein folding, DNA repair processes, endoplasmic reticulum (ER) stress, inflammation, autophagy, and/or apoptosis. Similar cellular phenotypes are frequently observed in other human diseases^31–33^. The source and physiological consequences of the multiple trace metals (e.g. titanium, nickel, strontium) found in appendicoliths is unknown.

Fourth, and perhaps most important, we found that appendicoliths contain identifiable human and bacterial proteins. Enrichment analysis of the human proteins that were common to the appendicoliths studied showed antioxidant activity and a variety of neutrophil pathways to be of importance. Interestingly, our analysis showed appendicoliths contain relatively high concentrations of several S100 calcium-binding proteins (S100A8/A9). In agreement with our findings for appendicoliths, a prior gene array study of expression in the inflamed appendix found S100A8 and S100A9 to be highly upregulated^34^. S100 calcium-binding proteins are abundant in neutrophils, are well-known pro-inflammatory markers and previously have been proposed as diagnostic serum biomarkers for appendicitis^35,36^. Higher fecal levels of these proteins have also been correlated with the severity of disease in inflammatory bowel disease^37,38^. Whether the antioxidant activity and activation of neutrophil pathways relate to dietary factors (e.g. fatty acids, food additives), redox-active metals, changes in the microbiome, or some combination of these is unknown, but our protein data clearly implicate the involvement of neutrophils, a rich source of reactive oxygen species, in this condition.

**Figure 4:**
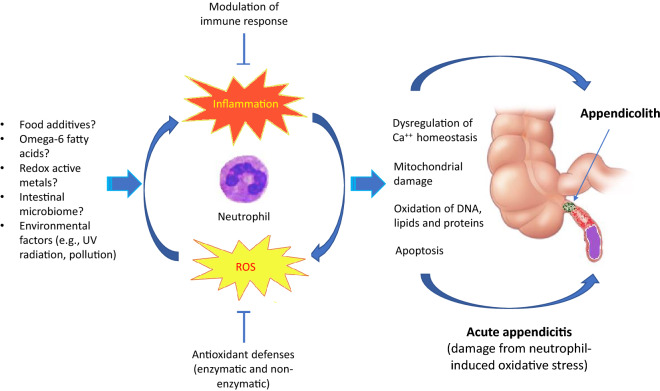
Conceptual model of the proposed relationship between oxidative stress and the formation of appendicoliths and acute appendicitis. See text for details.

Considered together, our findings suggest that oxidative stress has a role in the formation of appendicoliths and possibly in the etiology of acute appendicitis (Fig. 4). This hypothesis is a synthesis of our protein mass spectroscopy data showing enrichment of neutrophil activation and antioxidant pathways within appendicoliths, identification of substances known to cause oxidative stress (fatty acids and redox-active metals), and the existing literature on the biological consequences of oxidative stress on cellular function. Oxidative stress can cause mitochondrial damage, oxidation of DNA, lipids, and proteins, and dysregulation of calcium homeostasis^39,40^, the latter of which might explain the accumulation of calcium and phosphorus in appendicoliths. Based on our findings we propose that acute appendicitis occurs when neutrophil-induced oxidative stress exceeds the capacity of appendix antioxidant defenses to protect against reactive oxygen species. If not recognized and treated promptly, the resulting tissue damage can progress to necrosis and perforation of the appendix. We have identified the neutrophil as a key cell type involved in this pathology, which is consistent with the observation that infiltration of neutrophils is the hallmark of the histopathological diagnosis of acute appendicitis^41^. Further, our oxidative stress model can potentially explain several recent, seemingly unrelated observations on acute appendicitis. First, two genome-wide association studies (GWAS) have shown appendicitis to be associated with the rs2129979 locus at 4q25^42,43^. The gene nearest the association signal is *PITX2*, which encodes the transcription factor Paired-Like Homeodomain 2. The *PITX2* gene has a role in intestinal development^44–46^ but postnatally may mediate oxidative stress^47–49^. Second, the use of metformin, an antidiabetic drug, is associated with a reduced risk of appendicitis^50^. Metformin is thought to exert anti-inflammatory and antioxidant effects^51^. Third, low mitochondrial DNA (mtDNA) copy number and reduced mtDNA integrity, as evidenced by the formation of 8-hydroxyl-20-deoxyguanosine (8-OHdG), have been demonstrated in severe appendicitis^52^. The amount of 8-OHdG accumulated in mtDNA is considered an index of cellular oxidative damage^53^.

Our study has some limitations. Foremost is that the number of appendicoliths analyzed was small. Despite the small number, we have confidence in our results as the major findings were consistent across all samples. Another limitation is that appendicolith specimens were collected from pediatric patients. It is possible that samples from adults might have yielded different results. In addition, we are uncertain the extent to which our findings are due to mechanical trauma to the appendiceal mucosa from the appendicolith, or if our pathway findings relate solely to the inflammation associated with acute appendicitis. Finally, we did not analyze bacterial proteins found within the appendicoliths. With interest in the role of the intestinal microbiome in the pathogenesis of acute appendicitis growing^54–56^, this would be a fertile area for future research.

In conclusion, using state-of-the-art analytical techniques we show that appendicoliths have unique biochemical properties. These biological properties when considered together suggest oxidative stress may have a role in the formation of appendicoliths. Future work is needed to confirm these preliminary findings and to better understand how dietary factors such as food additives, omega-6 fatty acids, redox-active metals and the intestinal microbiome contribute to the oxidative load within the appendix. Understanding the complex interplay that exists between these environmental factors and genetics could provide important insight into appendicolith formation and the etiology of acute appendicitis, and ultimately lead to novel prevention strategies for this common disease.

## Supplementary Information

Supplementary Tables.

## Data Availability

The datasets used and/or analyzed during the current study are available from the corresponding author on reasonable request.
